# Geographic Heterogeneity in Influenza and Pneumonia Mortality in Hispanic Americans

**DOI:** 10.3390/ijerph18094917

**Published:** 2021-05-05

**Authors:** Annika Diaz-Campbell, Mahbubur Sumon, Alem Mehari, Mackenzie B. Snead, Rafael Ramirez, Elizabeth Arend, Richard F. Gillum

**Affiliations:** 1 Howard University College of Medicine, Washington, DC 20059, USA; annika.campbell@bison.howard.edu (A.D.-C.); mackenzie.snead@bison.howard.edu (M.B.S.); 2 Division of Pulmonary Medicine and Critical Care, Howard University Hospital, Washington, DC 20060, USA; MSumon@huhosp.org; 3 Department of Medicine, Howard University College of Medicine, Washington, DC 20059, USA; alem.mehari@howard.edu; 4 Primary Care Coalition of Montgomery County, Silver Spring, MD 20910, USA; Rafael_Ramirez@primarycarecoalition.org (R.R.); Elizabeth_Arend@primarycarecoalition.org (E.A.)

**Keywords:** influenza, pneumonia, mortality, Hispanic, geography, urbanization

## Abstract

(1) *Background*: Influenza and pneumonia (IP) is a leading cause of death in the US. The hypothesis was tested that the mortality rate differential between Hispanic whites (HW) and non-Hispanic whites (NHW) from IP varied by geographic region in the US. (2) *Methods*: The CDC database for multiple causes of death between 1999–2018 was used for this study. For ages 25–84, age-adjusted mortality rates per 100,000 (AAMR) for IP were computed by Hispanic ethnicity in whites for 10 Health & Human Services (HHS) regions and for urbanization levels in HHS Region 2. (3) *Results*: AAMR for IP was 13.76 (13.62–13.9) in HW and 14.91 (14.86–14.95) in NHW (rate ratio 1.08). Among HHS regions, rates were generally lower in HW than in NHW with the major exception of HHS Region 2. The rate there was 21.78 (21.24–22.33) in HW, 36.5% greater (*p* < 0.05) than that in NHW of 15.71 (15.56–15.86). In large central metro areas of Region 2, the rate was 27.10 (26.36–27.83) in HW compared to 19.78 (19.47–20.09) in NHW. (4) *Conclusion*: The difference in AAMR from IP between HW and NHW varied by region and urbanization with much higher rates for HW than NHW only in metropolitan areas of New York and New Jersey.

## 1. Introduction

Influenza and pneumonia (IP) is a leading cause of death in the US. In published reports, Hispanics had lower age-adjusted mortality rates (AAMR) per 100,000 compared to non-Hispanic whites (NHW) for every year since 1985. For example, in 1985, the AAMR for Hispanics was 30.2 compared to 34.3 for NHW; in 2000, the AAMR for Hispanics was 20.6 compared to 23.5 for NHW; in 2017, the AAMR for Hispanics was 11.3 compared to 14.2 for NHW [1]. A report of in-hospital mortality from pneumonia in 2005–2006 suggested a similar finding with an odds ratio (OR) of 0.85 with a 95% confidence interval (CI) of 0.81–0.89 compared to NHW in the same hospitals [2]. Considerable variation in mortality rates among US regions has been observed but geographic variation in ethnic disparities has been little studied [3].

Data from the US Vital Statistics System were analyzed to test the hypothesis that the mortality rate differential between Hispanic whites (HW) and NHW varied by geographic region in the US between 1999 and 2018.

## 2. Materials and Methods

### 2.1. Population and Deaths

This study used the Centers for Disease Control and Prevention (CDC) database for multiple causes of death (MCOD) between 1999–2018 [4]. Diagnostic data from death certificates were coded, including the underlying cause of death (UCOD), with up to 20 contributing causes of death. The UCOD is defined as the chief reason for death identified on the death certificate. The data used are publicly available and use does not constitute research with human subjects according to title 45, part 45 of the Code of Federal Regulations as data were de-identified and of aggregate nature. 

The total number of decedents with International Statistical Classification of Diseases and Related Health Problems, 10th revision (ICD-10), codes for influenza (J09–J11) and pneumonia (J12–J18) in the MCOD database were identified for 1999–2018. All pneumonia etiologies were included. Decedents’ ethnicity was coded as either non-Hispanic or Hispanic. Hispanic origin was not reported on the death certificate for some deaths (0.3%). On the mortality file, missing Hispanic origin information was coded as “not stated.” There was no corresponding population figure for this group. Therefore, deaths with Hispanic origin not stated were excluded when death rates were calculated by Hispanic origin. Table S1 shows total IP deaths by Hispanic origin in 1999–2018. Analyses were restricted to the white race category (90% of Hispanics) [4].

### 2.2. Regions and Urbanization

AAMR were grouped by Health & Human Services (HHS) regions to assess geographic variation in racial disparity of IP as an UCOD. See Table S2 for definitions of regions. 

Special analyses are published on occasion for classifying urbanization levels. The 2013 National Center for Health Statistics (NCHS) Urban-Rural Classification Scheme defined metropolitan counties for years as follows:

Large central metro counties are counties in metropolitan statistical areas (MSA) of 1 million or more population that (1) contain the entire population of the largest principal city of the MSA, (2) are completely contained in the largest principal city of the MSA, or (3) contain at least 250,000 residents of any principal city of the MSA. Large fringe metro counties are counties in MSAs of 1 million or more population that do not qualify as large central. The remaining categories are shown in Table S3 [5]. 

Analysis of IP-related mortality in HW and NHW by urbanization was limited to the entire US and to HHS Region 2. Use of the previous 2006 scheme yielded similar results; see Table S4.

### 2.3. Immunization

Data on the prevalence of immunization against pneumococcal disease and influenza were obtained from national surveys conducted by the CDC in selected years between 1999–2018 [6]. Data sources included the Behavioral Risk-Factor Surveillance System (BRFSS), the National Health Interview Survey (NHIS), and the National Immunization Survey-Flu (NIS-Flu). For example, the questions from the NHIS in 2015 include: “During the past 12 months, have you had a flu shot? A flu shot is usually given in the fall and protects against influenza for the flu season” [7]. “Have you ever had a pneumonia shot? This shot is usually given only once or twice in a person’s lifetime and is different from the flu shot. It is also called the pneumococcal vaccine”. Data for HW were not available, so data for all Hispanics were compared to NHW. Differences in immunization rates between Hispanics and NHW were calculated as percent immunized in NHW minus percent in Hispanics.

### 2.4. Analysis

The crude death rate by race and ten-year age groups was computed for decedents age 25 years and older. For ages 25–84, AAMR per 100,000 were computed using the 2000 US standard population as recommended by the CDC for all studies using CDC data [8]. The 95% CI was calculated for decedents at time of death for all analyses [9]. Mortality rates were considered significantly different if their 95% CI did not overlap. 

## 3. Results

### 3.1. Ethnicity and Death Rates

Among 28,547,345 deaths from all causes in HW and NHW from 1999–2018, Hispanic ethnicity was not stated in 75,990 (0.27%). For all causes of death, the AAMR was 30% higher in NHW than in HW (see Figure S1). 

**Figure 1:**
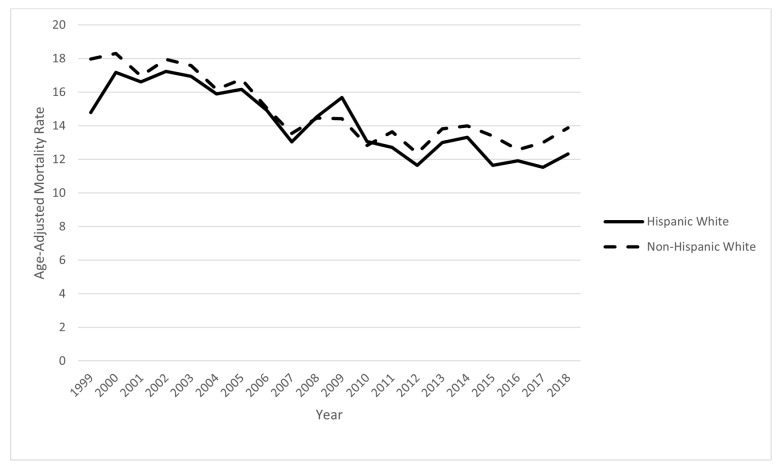
Trends in age-adjusted mortality rate for influenza/pneumonia: US 1999–2018.

For all years 1999–2018 combined, there were 136,321 mentions of IP on death certificates for HW ages 25–64. Of these, 39,131 (28.7%) were coded as UCOD. IP was mentioned on 1,700,144 NHW death certificates with 466,230 (27.4%) including IP as an UCOD. The AAMR per 100,000 for MCOD was 46.93 (95% CI 46.67–47.19) in HW and 54.04 (53.96–54.12) in NHW (rate ratio 1.15). The AAMR for UCOD was 13.76 (13.62–13.9) in HW and 14.91 (14.86–14.95) in NHW (rate ratio 1.08). Influenza was the cause of only 6.4% of IP-related deaths in HW and 4.9% in NHW. All following analyses are for UCOD. AAMRs declined over the period 1999–2018 in both HW and NHW, as shown in Figure 1. In 2018, AAMRs (95% CI) were HW 12.35 (11.86–12.84) and NHW 13.92 (13.74–14.09). Rates in HW were lower than in NHW in many but not all years.

Examination of ten-year age groups revealed that crude rates of IP did not differ significantly at ages 55–64 and 65–74 (Table 1). Among those aged 85 years and older, crude rates of IP were much higher in NHW than HW: 519 (517.5–520.4) vs. 403 (397.5–408.4) per 100,000. This was likely due in part to confounding by age in this open-ended age group. The AAMR were also examined for working-age persons aged 25–64 and older persons aged 65–84. For IP, the AAMR in NHW was 8.35% higher than HW for those aged 25–84, 11.65% higher for NHW aged 25–64, and 7.46% higher for NHW aged 65–84.

### 3.2. Regions and Urbanization

**Figure 2:**
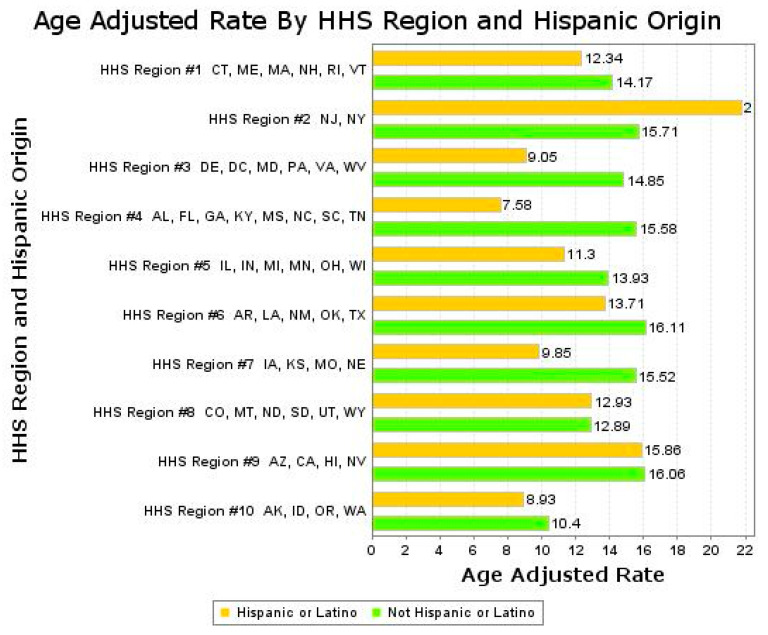
Age-adjusted mortality rate for influenza/pneumonia by Hispanic origin and HHS Region. X-axis units are deaths per 100,000.

Figure 2 and Table S5 show the AAMR for IP as a UCOD by HHS region for HW and NHW for 1999–2018 combined. Rates were generally lower in HW than in NHW with the major exception of HHS Region 2 (New York and New Jersey). The IP-related AAMR there was 21.78 (21.24–22.33) in HW, 36.5% greater (*p* < 0.05) than the NHW AAMR of 15.71 (15.56–15.86). The IP-related AAMR for HW in HHS Region 2 is the highest in any group or region, whereas the IP-related AAMR of HW in HHS Region 4 was the lowest (7.33).

From 1999–2018, the AAMR for IP was lower in HW than in NHW in each of the six urbanization strata (Figure S1). To further examine Region 2, AAMR were computed for HW and NHW by urbanization (Table 2). The vast majority of the 6546 IP-related deaths in HW occurred in large central metro areas (5464); the AAMR was 27.1 (26.36–27.83) for HW compared to 19.78 (19.47–20.09) for NHW. The AAMR was much lower in large fringe metro areas and less urbanized areas. In HHS Region 2, the percentage of IP deaths due to influenza was 2.5% in HW and 2.6% in NHW.

### 3.3. Immunization

**Figure 3:**
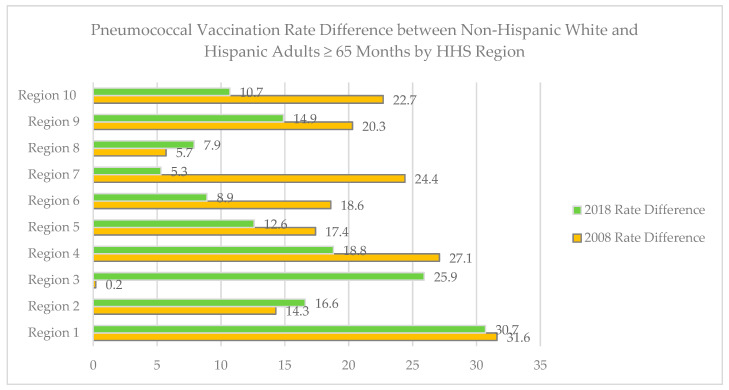
Difference in pneumococcal immunization rate between non-Hispanic white and Hispanic adults aged ≥ 65 years by HHS Region in 2008 and 2018 (Source BRFSS). X-axis units are percentages.

Geographic patterns in disparities in rates of immunization among HW and NHW were examined in national data. National pneumococcal immunization rates for individuals aged 65 years and older were substantially lower in all Hispanics than NHW from 1999–2018, e.g., in 2008, Hispanics 49.9%, NHW 69.1%; in 2018, Hispanics 59.9% vs. NHW 74.9%. Figure 3 shows the difference in percentage points between Hispanics and NHW among HHS divisions in 2008 and 2018. In 2008, Hispanics had substantially lower pneumococcal immunization rates except in Regions 3 (Mid-Atlantic) and 8 (Mountain). In 2018, differences were smallest in Regions 6, 7, and 8.

Similarly, national influenza immunization rates for individuals aged 18 and older were substantially lower in all Hispanics than NHW from 1999–2018 (Figure S2). However, for both Hispanics and NHW, rates at 65 years and older were lower in New York and New Jersey than in most other regions (Figure S3). Within New York State, immunization rates declined between 2013 and 2018 but were similar between Hispanics and NHW (Figure S4).

## 4. Discussion

This analysis of US vital statistics showed that the difference in AAMR from IP between HW and NHW varied by HHS region and urbanization but were generally lower in HW than NHW. However, the extremely high IP-related AAMR for HW in Region 2 (New York and New Jersey) exceeded those of NHW, mostly due to rates in large central metropolitan areas. Disparities in IP immunization did not seem to contribute to the disparity in Region 2 or to the patterns elsewhere.

### 4.1. Previous Studies

Although relatively few deaths are coded as due to influenza, vital statistics published by the CDC combine influenza and pneumonia because influenza can lead to pneumonia. The combined category has long been among the top ten causes of mortality in the US [1]. These statistics showed a decades-long pattern of lower IP-related AAMR in HW than NHW. Relatively few population-based studies have examined pneumonia mortality among Hispanics. Using national hospital data provided by the Centers for Medicare and Medicaid (CMS) on over 1 million pneumonia admissions in 2005–2006, one study found a lower in-hospital death rate in Hispanics compared to NHW [2]. This difference was seen in the same hospitals after adjustment for age and other variables (OR = 0.85, 95% CI 0.81–0.89), consistent with the Hispanic mortality paradox [2]. An examination of quality indicators showed that Hispanics were less likely than NHW to receive their first antibiotic dose within four hours (71% vs. 78%), pneumonia vaccination (53% vs. 68%), or smoking counseling (76% vs. 82%). Further analyses indicated that more of these disparities in quality were due to attending low-performing hospitals rather than within-hospital ethnic differences [2].

The phenomenon of lower total or cause-specific mortality rates in Hispanic immigrants in the US compared to US-born persons or all persons in the US has been referred to as the “Hispanic mortality paradox” [10]. It is considered a paradox because Hispanic immigrants from Latin America are more likely to have lower education, income, and occupational status than non-Hispanic individuals born in the US. These variables are generally associated with higher mortality rates. Although this phenomenon has been observed in a number of studies, other studies failed to detect the Hispanic mortality paradox as extensively reviewed elsewhere [10]. Data artifact is one possible explanation. When that is excluded, a number of biological and psychosocial theories have been advanced to explain it and factors modifying any effect identified [10]. A 2017 meta-analysis of 28 studies of mortality among immigrants from Latin America and the Caribbean found a significant advantage for immigrants compared to US-born persons for all causes and cardiovascular mortality for persons aged 20–64 years but not for those 65 years and older [10].

To what extent are the findings of the present study consistent with the Hispanic mortality paradox? A comparison of US AAMR due to IP at all ages in 1985–2018 is consistent with the phenomenon, as is the above-cited study of US hospital mortality [1,2]. Despite being served more often in lower-performing hospitals, HW still had lower AAMR. Central metropolitan residence and insurance status (e.g., Medicaid, self-pay) often determine usage of such facilities by HW [11,12]. 

The much higher AAMR in HW than NHW in HHS Region 2 (NY, NJ) is not consistent with the Hispanic mortality paradox, however. This indicates that factors must exist there that overwhelm any protective factors that produce lower rates in HW than NHW in other regions. Some insight into this deviation may be derived from a closer look at New York City (NYC), the largest metropolitan area in Region 2 with a Hispanic population of approximately 2.5 million individuals (29% of the population) [13]. In contrast to the US, IP is the third leading cause of death in NYC [12]. A geospatial analysis of pneumonia hospitalization was done in 188 neighborhood tabulation areas (NTA) in 2004–2006 [12]. In clusters of NTA with high IP age-adjusted hospitalization rates, 29.4% of patients were Hispanic [12]. In clusters of NTA with low IP rates, only 5.0% of patients were Hispanic [12]. Areas with high IP rates, such as parts of the Bronx, also had high rates of poverty and chronic disease [14]. 

Hispanics from Puerto Rico have been found to have higher chronic disease rates than other Hispanic groups [15,16]. Moreover, previous studies indicate a significant increase in all-cause mortality rates and AAMR due to IP for Puerto Rican populations as compared to other Hispanic groups [15]. An analysis of mortality in 2009 showed a higher AAMR of 17.7 (95% CI 16.0–19.4) for IP for Puerto Ricans living in the continental US compared to the AAMR for IP among NHW, 16.2 (15.9–16.2) [15]. Puerto Ricans comprise 29% of the Hispanic population in NYC which does not completely explain the deviation in AAMR for Region 2 [13]. The Hispanic Community Health Study/Study of Latinos (HCHS/SOL) also found that Puerto Ricans had a higher prevalence of cigarette smoking and lower IP vaccination rates than other Hispanic groups [17].

Prior reports of IP immunization do not explain lower IP-related AAMR in HW than NHW. Over decades, Hispanics were found to have lower immunization rates than NHW [7,18,19]. In NYC, in 2008, only 41% of Hispanics aged 65 and older received the pneumococcal vaccination as compared to 54% of NHW [13,20]. Influenza vaccination rates were also lower in Hispanics than in NHW. Future studies should assess geographic variation in ethnic disparities in immunization between Hispanics of differing national origins and places of birth vs. NHW and origin of the excess mortality from COVID-19 as compared to IP in HW.

### 4.2. Strengths and Limitations

Strengths of this report include the use of high-quality vital statistics data from the CDC with large numbers of deaths. ICD-10 was in use throughout the study period, so changes do not affect trends shown in Figure 1. Limitations include the well-described systematic underestimates of Hispanic death rates by about 5% due to misclassification of ethnicity on death certificates [21]. Geographic variation in physician diagnostic and death certificate coding practices cannot be excluded. Analyses by Hispanic subgroup were not possible with the analysis system used [4]. Future research should examine Hispanic subgroups and correlate geographic patterns and trends with absolute and relative numbers of Hispanics in the population.

## 5. Conclusions

In this analysis of data from 1999–2018, the difference in AAMR from IP between HW and NHW varied by region and urbanization. The AAMR from IP in HW greatly exceeded those in NHW only in HHS Region 2 (NY and NJ).

## Figures and Tables

**Table 1:** Influenza and pneumonia deaths and crude mortality rate per 100,000 by age group for Hispanic whites and non-Hispanic whites, United States: 1999 to 2018.

Age Groups (Years)	Hispanic White			Non-Hispanic White		
	Deaths	Crude Rate	Crude Rate 95% CI ^1^	Deaths	Crude Rate	Crude Rate 95% CI
25–34	1183	0.8	0.8–0.9	4747	1.0	0.9–1.0
35–44	2113	1.7	1.6–1.7	11,249	2.1	2.0–2.1
45–54	3716	4.0	3.9–4.1	27,723	4.7	4.6–4.7
55–64	5866	10.4	10.1–10.6	54,949	10.7	10.6–10.8
65–74	9516	30.7	30.1–31.3	106,432	30.3	30.2–30.5
75–84	16,737	107.6	106.0–109.3	261,130	119.6	119.1–120.1
85+	21,060	403.0	397.5–408.4	465,710	518.9	517.5–520.4

^1^ Confidence Interval.

**Table 2:** Influenza and pneumonia deaths and age-adjusted mortality rate per 100,000 by urbanization for Hispanic whites and non-Hispanic whites in HHS Region 2 (New York and New Jersey).

2013 Urbanization	Hispanic White			Non-Hispanic White		
	Deaths	AAMR ^1^	AAMR 95% CI ^2^	Deaths	AAMR	AAMR 95% CI
Large Central Metro	5464	27.10	26.36–27.83	16,090	19.78	19.47–20.09
Large Fringe Metro	900	10.46	9.73–11.18	17,227	13.35	13.15–13.55
Medium Metro	86	11.92	9.36–14.96	4763	14.91	14.48–15.33
Small Metro	49	12.70	9.22–17.04	2322	15.39	14.76–16.02
Micropolitan (Nonmetro)	31	21.04	13.98–30.41	2357	15.94	15.29–16.58
Non-Core (Non-metro)	16	Unreliable	7.88–25.32	932	15.24	14.25–16.23

^1^ Age-adjusted mortality rate (AAMR). ^2^ Confidence interval.

## Data Availability

Data available in a publicly accessible repository that does not issue DOIs Publicly available datasets were analyzed in this study. This data can be found here: https://wonder.cdc.gov/mcd.html (accessed on 4 May 2021).

## Supplementary Materials

The following are available online at https://www.mdpi.com/article/10.3390/ijerph18094917/s1, Table S1: IP deaths by ethnicity; Table S2: States and territories in regions designated by the Department of Health and Humans Service (HHS); Table S3: Definitions of urbanization levels from 2013 NCHS Urban-Rural Classification Scheme; Table S4: Influenza and pneumonia deaths and age-adjusted mortality rate per 100,000 by urbanization for Hispanic whites and non-Hispanic whites in HHS Region 2, 2006 NCHS Urban-Rural Classification Scheme; Table S5: Deaths from all causes and age-adjusted mortality rate (AAMR) per 100,000 of death from influenza and pneumonia by HHS Region and Hispanic ethnicity; Figure S1: Nationwide age-adjusted mortality rate (AAMR) of influenza and pneumonia per 100,000 by 2013 urbanization type for Hispanic whites and non-Hispanic whites; Figure S2: Up-to-date influenza immunization rate by race/ethnicity in adults ≥ 18 years: United States, Behavioral Risk Factor Surveillance System (BRFSS), 2007–2020; Figure S3: Maps of adults aged 65+ who have had a flu shot within the past year; Figure S4: Percent of adults aged 65+ who received an influenza vaccination in New York State by year and race/ethnicity.
