# Supplementary material for: Geographic Heterogeneity in Influenza and Pneumonia Mortality in Hispanic Americans
Source: Int J Environ Res Public Health. 2021 May 5;18(9):4917. doi: 10.3390/ijerph18094917 (PMC8125250; doi:10.3390/ijerph18094917)
Supplement: Supplementary file 1 [file ijerph-18-04917-s001.zip › ijerph-1154063-supplementary/ijerph-1154063 Supplementary Metarials/Figure S3.pdf]

Topic: Flu Shot

Adults aged 65+ who have had a flu shot within the past year (variable calculated from one or more BRFSS questions)

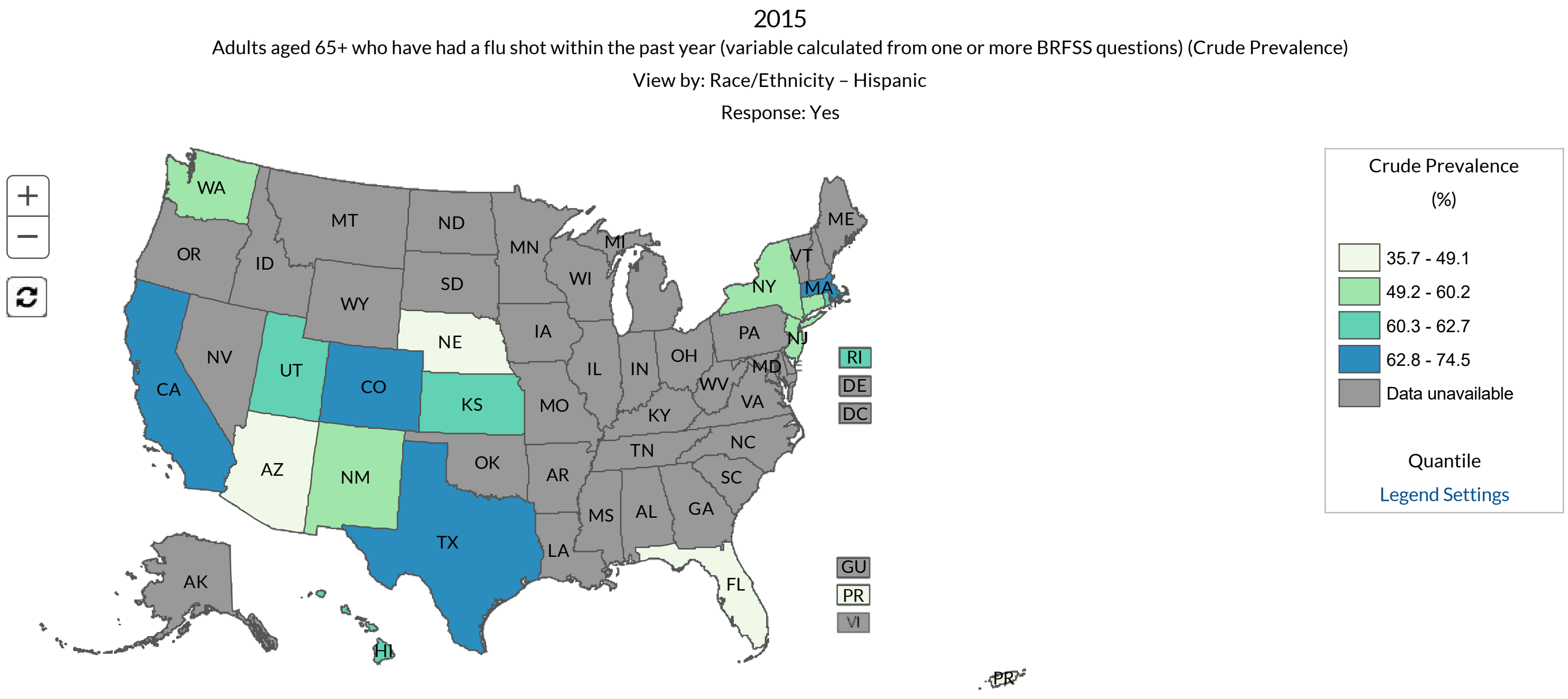

Footnote

* Prevalence estimate not available if the unweighted sample size for the denominator was < 50 or the Relative Standard Error (RSE) is > 0.3 or if the state did not collect data for that calendar year.

Data Source: Behavioral Risk Factor Surveillance System (BRFSS)

Note

Use caution in interpreting cell sizes less than 50.

If you see that a question is not available for a particular year, it is because the question was dropped or changed. Check the category of interest for that year to find similar items.

Denominator includes all respondents except those with missing, don't know, and refused answers.

** Median value reported with no confidence intervals.

Measure Definitions

% = Percent

CI = 95% Confidence Interval

n = “Sample Size”. Translates to the number of adult respondents who answered this question, or were assigned to this category based on question responses for a calculated variable, or the number of states included for a median.

N/A = Translates to no data available for a record.

No Data = Translates to no data available for record.

Topic: Flu Shot

Adults aged 65+ who have had a flu shot within the past year (variable calculated from one or more BRFSS questions)

2015

Adults aged 65+ who have had a flu shot within the past year (variable calculated from one or more BRFSS questions) (Crude Prevalence)

View by: Race/Ethnicity – White, non-Hispanic

Response: Yes

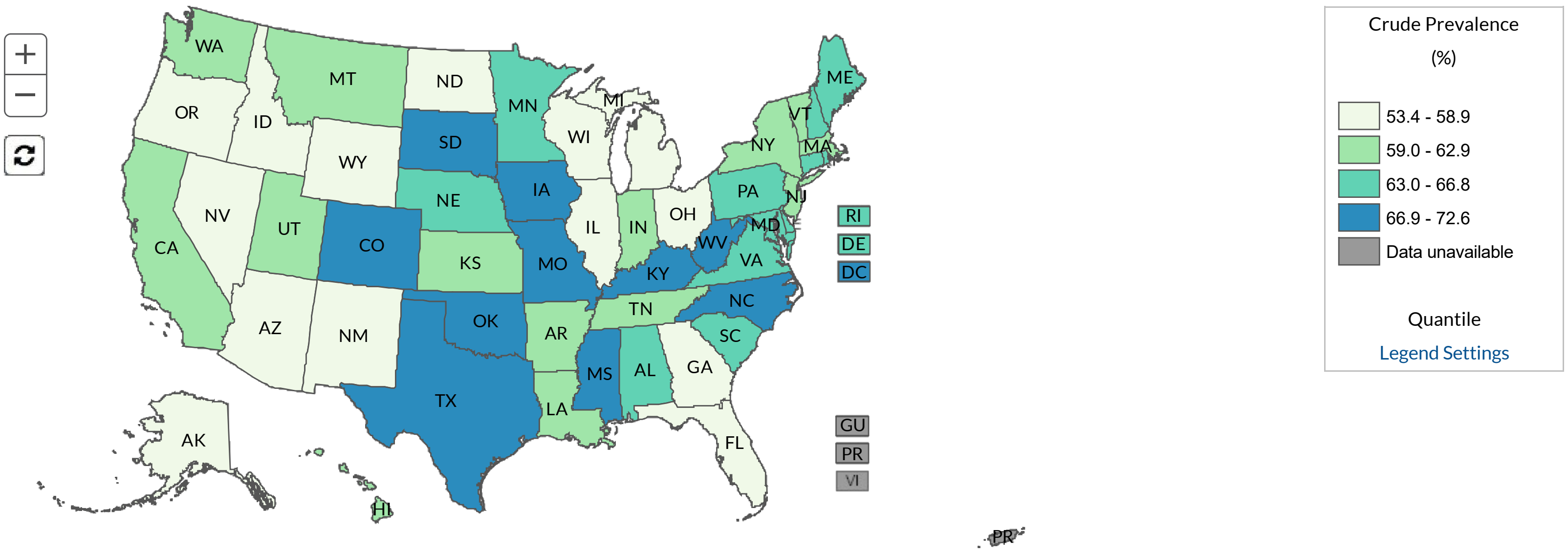

Footnote

* Prevalence estimate not available if the unweighted sample size for the denominator was < 50 or the Relative Standard Error (RSE) is > 0.3 or if the state did not collect data for that calendar year.

Data Source: Behavioral Risk Factor Surveillance System (BRFSS)

Note

Use caution in interpreting cell sizes less than 50.

If you see that a question is not available for a particular year, it is because the question was dropped or changed. Check the category of interest for that year to find similar items.

Denominator includes all respondents except those with missing, don't know, and refused answers.

** Median value reported with no confidence intervals.

Measure Definitions

% = Percent

CI = 95% Confidence Interval

n = “Sample Size”. Translates to the number of adult respondents who answered this question, or were assigned to this category based on question responses for a calculated variable, or the number of states included for a median.

N/A = Translates to no data available for a record.

No Data = Translates to no data available for record.

Centers for Disease Control and Prevention, National Center for Chronic Disease Prevention and Health Promotion, Division of Population Health. BRFSS Prevalence & Trends Data [online]. 2015. [accessed Jan 12, 2021]. URL: https://www.cdc.gov/brfss/brfssprevalence/.
