# Supplementary material for: Geographic Heterogeneity in Influenza and Pneumonia Mortality in Hispanic Americans
Source: Int J Environ Res Public Health. 2021 May 5;18(9):4917. doi: 10.3390/ijerph18094917 (PMC8125250; doi:10.3390/ijerph18094917)
Supplement: Supplementary file 1 [file ijerph-18-04917-s001.zip › ijerph-1154063-supplementary/ijerph-1154063 Supplementary Metarials/ijerph-1154063 Supplementary Metarials For Proof.pdf]

International Journal of
Environmental Research and Public Health

MDPI

Table S1. Number of deaths with influenza/pneumonia as an underlying cause of death by Hispanic ethnicity.

	Hispanic or Latino	Not Hispanic or Latino	Not Stated
Aged 25-64	13,230	132,110	769
Aged 65-84	26,840	429,018	1535
Female	18,014	26,3926	858
Male	22,056	297,202	1446
HHS Region #1	740	27,237	127
HHS Region #2	6847	56,931	790
HHS Region #3	733	60,498	188
HHS Region #4	3818	125,882	223
HHS Region #5	1920	95,730	382
HHS Region #6	9629	60,042	165
HHS Region #7	336	28,628	66
HHS Region #8	1031	15019	44
HHS Region #9	14,559	75,206	294
HHS Region #10	457	15,955	25

Table S2. States and Territories in regions designated by the Department of Health and Humans Service (HHS).

HHS Region	States and Territories
1	Massachusetts, Maine, New Hampshire, Rhode Island, Vermont, and Connecticut
2	New York, New Jersey
3	Maryland, Delaware, District of Columbia, Pennsylvania, West Virginia and Virginia
4	Tennessee, South Carolina, North Carolina, Mississippi, Kentucky, Georgia, Florida, and Alabama
5	Michigan, Minnesota, Illinois, Indiana, Wisconsin, and Ohio
6	Louisiana, Arkansas New Mexico, Texas, and Oklahoma
7	Missouri, Kansas, Iowa, and Nebraska
8	Montana, Colorado, South Dakota, North Dakota, Utah and Wyoming
9	Arizona, California, Hawaii, and Nevada
10	Washington, Oregon, Alaska, and Idaho

Table S3. Definitions of urbanization levels from 2013 NCHS Urban – Rural Classification Scheme.

2013 Urbanization	Definition
Large Central Metro	county in metropolitan statistical area (MSA) of greater than 1 million and (1)greater than 250,000 inhabitants from MSA's principal city, (2)county with whole population inside MSA's largest city, or (3) county with whole population of MSA's largest city
Large Fringe Metro	county in MSAs of 1 million or more population that do not qualify as Large Central Metro
Medium Metro	county in MSAs of 250,000 to 999,999 population
Small Metro	county in MSAs of less than 250,000 population
Micropolitan (Nonmetro)	county in a micropolitan statistical areas (i.e. with a principal city population of 10,000-49,000)
NonCore (Nonmetro)	county that is neither in a MSA nor a micropolitan statistical area

Table S4. Influenza and pneumonia deaths and age adjusted mortality rate per 100,000 by urbanization for Hispanic Whites and Non-Hispanic Whites in HHS Region 2, 2006 NCHS Urban – Rural Classification Scheme.

2006 Urbanization	Hispanic White				Non-Hispanic White			
	Deaths	AAMR [1]	AAMR 95% CI [2] Lower	AAMR 95% CI Upper	Deaths	AAMR	AAMR 95% CI Lower	AAMR 95% CI Upper
Large Central Metro	5355	27.82	27.06	28.59	15483	20.16	19.84	20.48
Large Fringe Metro	943	10.39	9.68	11.09	16538	13.11	12.91	13.31
Medium Metro	152	13.2	10.94	15.46	5998	15.45	15.05	15.84
Small Metro	48	12.75	9.27	17.12	2110	15.29	14.63	15.95
Micropolitan (Nonmetro)	32	20.51	13.63	29.64	2569	16.03	15.41	16.66
NonCore (Nonmetro)	16	Unreliable	7.67	24.64	993	15.26	14.3	16.22

[1] Age Adjusted Mortality Rate (AAMR). [2] Confidence Interval.

Table S5. Deaths from all causes and age adjusted mortality rate(AAMR) per 100,000 of death from influenza and pneumonia by HHS Region and Hispanic ethnicity.

HHS region	Hispanic White			Non-Hispanic White		
	Deaths	AAMR [a]	AAMR 95% CI [b]	Deaths	AAMR	AAMR 95% CI
HHS1	716	12.34	11.35–13.32	25858	14.17	13.99–14.34
HHS2	6546	21.78	21.24–22.33	43691	15.71	15.56–15.86
HHS3	704	9.05	8.32–9.78	50111	14.85	14.72–14.98
HHS4	3690	7.58	7.33–7.82	101833	15.58	15.48–15.67
HHS5	1868	11.3	10.75–11.86	82800	13.93	13.84–14.03
HHS6	9567	13.71	13.42–14	49141	16.11	15.97–16.26
HHS7	325	9.85	8.68–11.01	26621	15.52	15.33–15.71
HHS8	1003	12.93	12.08–13.78	13993	12.89	12.67–13.1
HHS9	14280	15.86	15.58–16.13	57538	16.06	15.92–16.19
HHS10	432	8.93	7.98–9.88	14644	10.4	10.23–10.57

[a]Age Adjusted Mortality Rate. [b]Confidence Interval.

Figure S1. Nationwide age adjusted mortality rate(AAMR) of influenza and pneumonia per 100,000 by 2013 urbanization type for Hispanic Whites and Non-Hispanic Whites

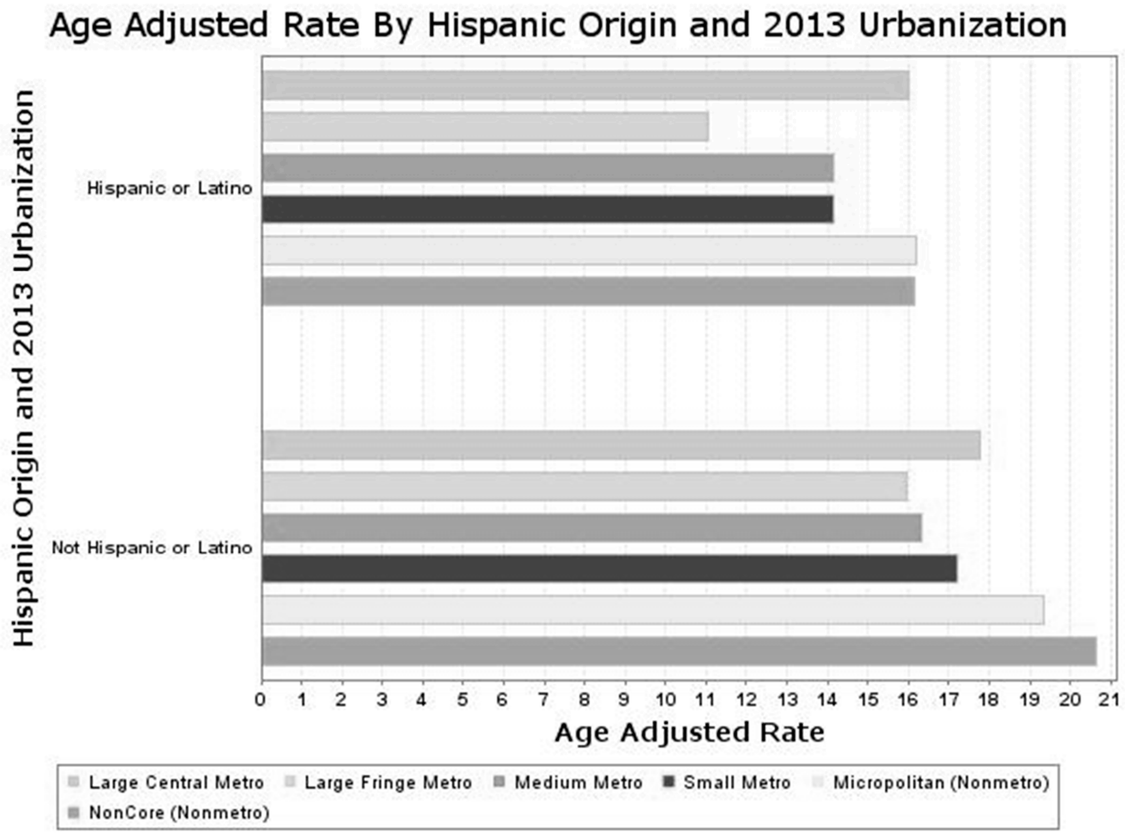

Figure S2. Up-to-date influenza immunization rate by race/ethnicity in adults ≥ 18 years: United States, Behavioral Risk Factor Surveillance System (BRFSS), 2007-2020.

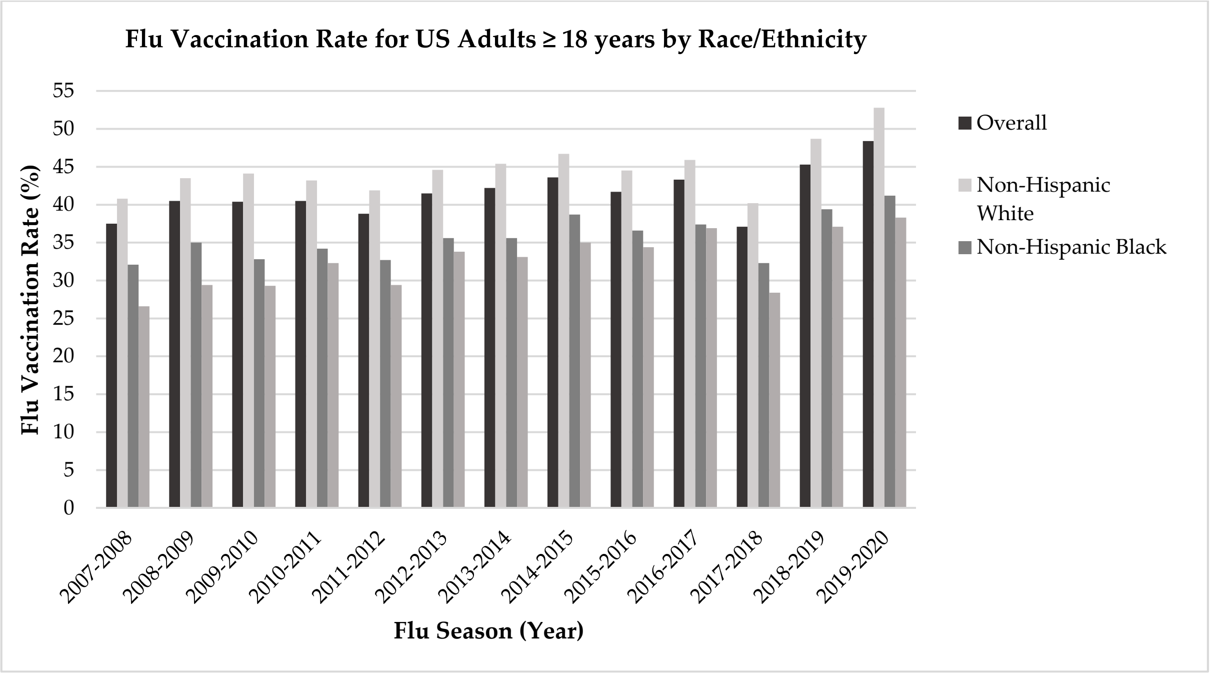

Data taken from BRFSS

Figure S4. Percent of Adults aged 65+ who received an influenza vaccination in New York State by Year and Race / Ethnicity.

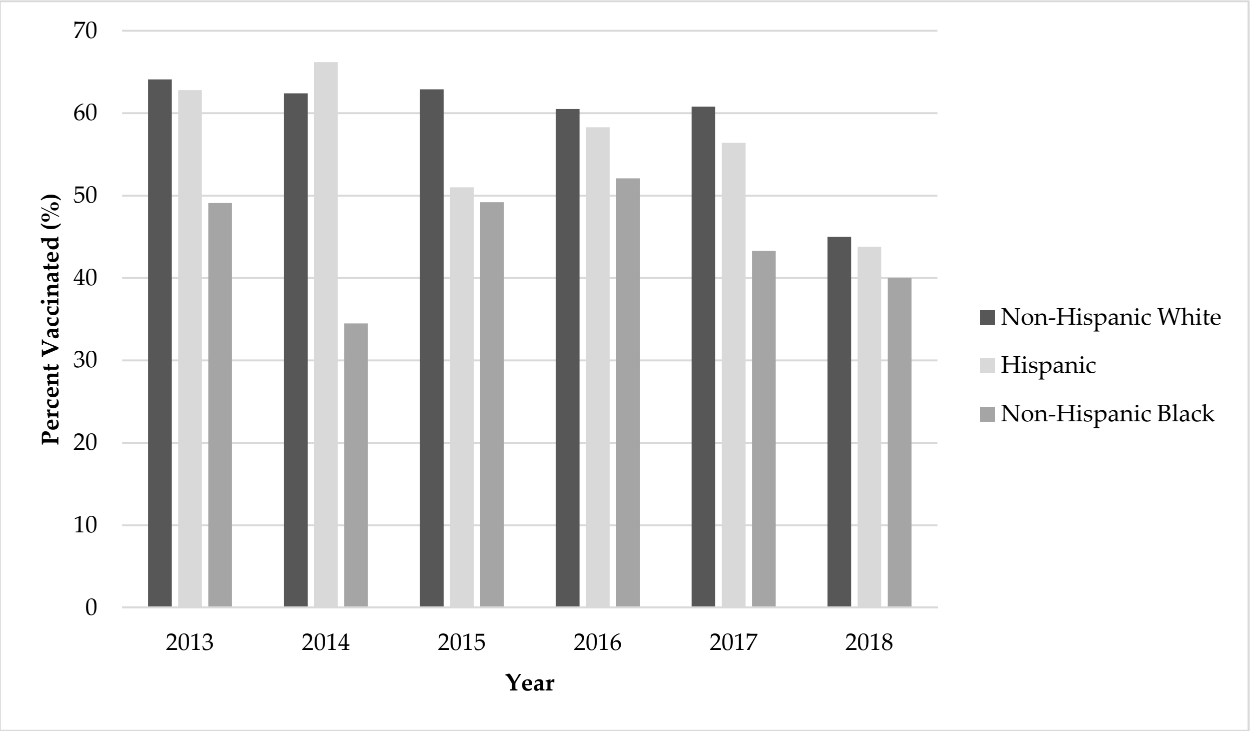

Data taken from BRFSS
